# Rationalizing artemisinin-based combination therapies use for treatment of uncomplicated malaria: A situation analysis in health facilities and private pharmacies of Douala 5^e^-Cameroon

**DOI:** 10.1371/journal.pone.0299517

**Published:** 2024-05-07

**Authors:** Carole Else Eboumbou Moukoko, Josiane Etang, Loick Pradel Kojom Foko, Christian Donald Tafock, Patricia Epee Eboumbou, Estelle Géraldine Essangui Same, Ida Calixte Penda, Albert Same Ekobo

**Affiliations:** 1 Department of Biological Sciences, Faculty of Medicine and Pharmaceutical Sciences, The University of Douala, Douala, Cameroon; 2 Malaria Research Unit, Centre Pasteur Cameroon, Yaoundé, Cameroon; 3 Laboratory of Parasitology, Mycology and Virology, Postgraduate Training Unit for Health Sciences, Postgraduate School for Pure and Applied Sciences, The University of Douala, Douala, Cameroon; 4 Yaoundé Research Institut, Organisation de Coordination pour la Lutte Contre les Endémies en Afrique Centrale (OCEAC), Yaoundé, Cameroon; 5 Department of Animal Biology, Faculty of Science, The University of Douala, Douala, Cameroon; 6 Department of Pharmaceutical Sciences, Faculty of Medicine and Pharmaceutical Sciences, The University of Douala, Douala, Cameroon; 7 Clinical Sciences Department, Faculty of Medicine and Pharmaceutical Sciences, University of Douala, Douala, Cameroon; 8 Pediatric wards, Bonassama Hospital, Douala, Cameroon; 9 National Roll Back Malaria Committee, Yaoundé, Cameroon; Shoklo Malaria Research Unit, THAILAND

## Abstract

Artemisinin-based combination therapies (ACTs) represent one of the mainstays of malaria control. Despite evidence of the risk of ACTs resistant infections in resource-limited countries, studies on the rational use of ACTs to inform interventions and prevent their emergence and/or spread are limited. The aim of this study was designed to analyze practices toward ACTs use for treating the treatment of uncomplicated malaria (UM) in an urban community. Between November 2015 and April 2016, a cross-sectional and prospective study was conducted in the 6 health facilities and all pharmacies in the Douala 5^e^ subdivision, Cameroon. Anonymous interviews including both open- and closed-ended questions were conducted with selected participants among drug prescribers, patients attending the health facilities, and customers visiting the pharmacies. Data analysis was performed using StataSE11 software (version 11 SE). A total of 41 prescribers were included in the study. All were aware of national treatment guidelines, but 37.7% reported not waiting for test results before prescribing an antimalarial drug, and the main reason being stock-outs at health facilities. Likewise, artemether+lumefantrine/AL (81%) and dihydroartemisinin+piperaquine (63.5%) were the most commonly used first- and second-line drugs respectively. Biological tests were requested in 99.2% (128/129) of patients in health facilities, 60.0% (74) were performed and 6.2% were rationally managed. Overall 266 (35%) of 760 customers purchased antimalarial drugs, of these, 261 (98.1%) agreed to participate and of these, 69.4% purchased antimalarial drugs without a prescription. ACTs accounted for 90.0% of antimalarials purchased from pharmacies, of which AL was the most commonly prescribed antimalarial drug (67.1%), and only 19.5% of patients were appropriately dispensed. The current data suggest a gap between the knowledge and practices of prescribers as well as patients and customers misconceptions regarding the use of ACTs in Douala 5^e^ subdivision. Despite government efforts to increase public awareness regarding the use of ACTs as first-line treatment for UM, our findings point out a critical need for the development, implementation and scaling-up of control strategies and continuing health education for better use of ACTs (prescription and dispensing) in Cameroon.

## Introduction

Malaria remains a public health problem in endemic areas and is caused mainly by five *Plasmodium* species *P*. *vivax*, *P*. *malariae*, *P*. *ovale spp*, *P*. *knowlesi* and *P*. *falciparum*, the most deadly species [1, 2]. Malaria kills more than half a million people each year, most of whom are children under the age of 5. In its latest report, the World Health Organization (WHO) pointed out that in 2021, malaria was responsible for 247 million cases of illness, including 409,000 deaths, worldwide. Sub-Saharan Africa (sSA) region continues to bear the highest burden, accounting for 95% of global cases and 93% of deaths [2].

Nonetheless, interventions prevented about 10.6 million malaria deaths between 2000 and 2020, mostly in Africa. Control methods such as long-lasting insecticide-treated nets and, insecticide residual spraying were responsible for most of the progress. But a significant number of lives have also been saved by artemisinin-based combination therapies (ACTs), which is used as a first-line treatment since their introduction in the early 2000s. Indeed, prompt and effective treatment is one of the mainstays of malaria control in endemic areas, and the implementation of ACTs along with complementary control methods contributed significantly to the reduction in the malaria burden between 2010 and 2019 [2].

ACTs are the current cornerstone of malaria treatment and consist of the combination of artemisinin (ART) (or one of its derivatives) with one of five partner drugs or drug combinations. When given together, the fast-acting artemisinin component kills most of the parasites within a few days, and the longer-acting partner drug clears the residual parasites.

Six ACTs are recommended by the WHO for the first- and second-line management of malaria; i) Artesunate + Amodiaquine (AS + AQ), ii) Artemether + Lumefantrine (AL), iii) Artesunate + Sulfadoxine-Pyrimethamine (AS + SP), iv) Artesunate + Mefloquine (AS + MQ), v) Dihydroartemisinin + Piperaquine (DHA + PPQ), and recently vi) Artesunate + Pyronaridine (AS + PY) [2]. These ACTs are variably adopted and used by malaria endemic countries.

Despite these tremendous gains, the rate of reduction in malaria mortality has slowed since 2016, while the decline in malaria incidence has remained at similar rates from 2014 to 2019 [2]. One of the reasons is the emergence and spread of *P*. *falciparum* isolates resistant to ACTs which are currently mainly localized in the Greater Mekong subregion (GMS), Southeast Asia [3, 4]. Over 200 nonsynonymous mutations in the propeller domain of *P*. *falciparum* kelch 13 (*pfk13*) gene have been identified worldwide (data on candidate and validated ART-resistance *pfk13* mutations are available at https://www.who.int/news-room/questions-and-answers/item/artemisinin-resistance) of which 22 were shown to be strongly involved in ART resistance [5], and to date twelve *pfk13* mutations (413A, 446I, 458Y, 469Y, 476I, 493H, 539T, 543T, 553L, 561H 574L, 580Y and 675V) have been validated to confer ART resistance [4–7].

The fear of seeing local resistance emerge or the spread of such resistance across Africa, which would be catastrophic because there is currently no immediate replacement for ACTs, is now becoming a reality [8, 9]. Recent published reports have confirmed that ACTs had failed to work quickly in more than 10% of participants at two sites in Rwanda [10–12], and the resistant malaria parasites had risen from 3.9%of cases in 2015 to nearly 20% in 2019 in Uganda [9, 13]. Genetic analysis shows that the resistance mutations in Rwanda and Uganda have emerged independently [14]. In addition, worrying signs of artemisinin resistance have been detected in Eritrea, as reported in the World Malaria Report 2022, but no peer-reviewed studies confirming such resistance have yet been published [15].

ACT resistance has severely hampered malaria cases management in the GMS [2]. In this context, the WHO launched the Global Plan for Artemisinin Resistance Containment in 2011 and, more recently, the “High Burden to High Impact” strategy, both aimed in part at protecting the efficacy of ACTs through improved access to diagnosis and rational treatment with ACTs [2, 16, 17]. The rational use of ACTs for uncomplicated malaria (UM) and severe malaria (SM) is essential to prevent the emergence of ACT-resistant parasites in endemic regions, especially in sSA, where ART resistance has been now been reported in two countries. In this context, it is of utmost importance to prevent the emergence through appropriate use of ACTs in this region.

Malaria is a major concern in Cameroon, where it accounts for 25–40% of medical consultations and 30–47% of hospitalizations [18]. Cameroon is among the 15 countries most affected by malaria, accounting for 2.9% of all malaria cases and deaths worldwide and 2.4% of malaria deaths in 2020 [18], making it the third most affected country in Central Africa (12.6% of cases in 2020). Current guidelines for first- and second-line treatment of UM in Cameroon are based on the use of ACTs [19, 20], while the biological diagnosis of malaria is mainly based on peripheral blood film (PBF) and rapid diagnostic tests (RDTs) [21, 22]. The few studies conducted in Cameroon to evaluate the rational use of ACTs reported frequent prescription of ACTs without biological confirmation of malaria infection and, in some case, under-prescription of ACTs (i.e., non-prescription of ACTs in patients with positive malaria RDT) [23–28]. In addition, Ndong *et al*. found that being male increased the odds of being prescribed ACTs regardless of the performance of biological confirmation in a health facility (HF) in the North West region of Cameroon [26]. A study in Cameroon (Yaoundé and Bamenda) and in Nigeria reported that health care workers’ prescription of ACTs was not related to their knowledge of national guidelines but to their personal preference. In addition, patient preferences, symptoms, previous antimalarial treatment and availability of ACTs may also influence the choice of ACTs by health care workers [29].

We were unable to identify in the literature any other population-based studies on the rational use of ACTs in Douala [24], the economic capital of Cameroon, despite being a crossroads and large city with high human population density and very high risk of malaria transmission [27]. Indeed, this study was based on interviews and prescription records to i) assess malaria case management and ii) identify determinants of the ACTs prescription and use in the Douala 5^e^ subdivision, in the Littoral Region of Cameroon.

## Materials and methods

### i) Study design and setting

**Fig 1:**
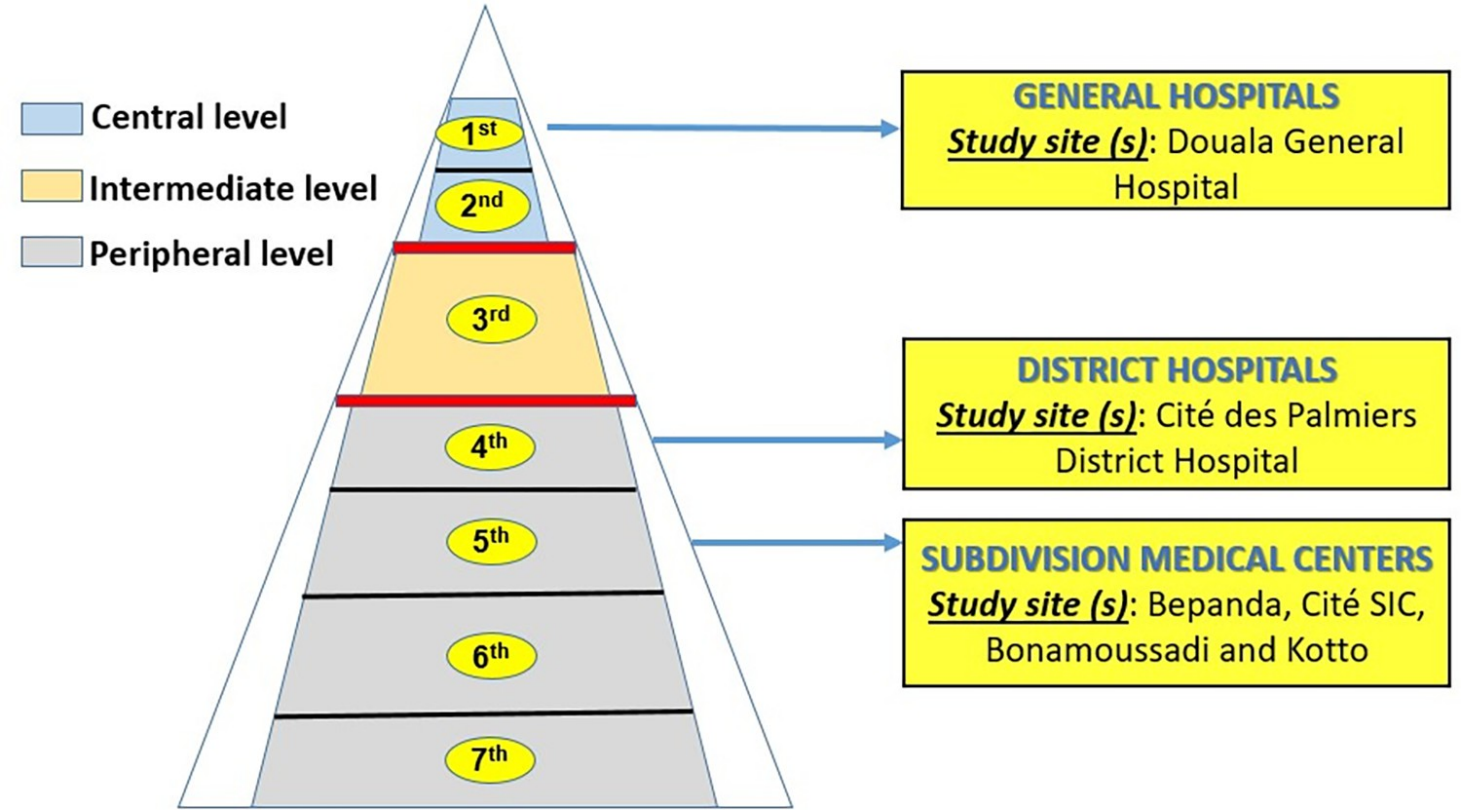
Pyramidal organization of health facility system in Cameroon. **Note.** Health facilities are of three levels (central, intermediate and peripheral) and categorized into 7^th^ categories viz. First category (General hospitals), second category (Central hospital), third category (Regional hospitals), fourth category (District hospital), fifth category (Subdivision medical centers), sixth category (Integrated health centers) and seventh category (Ambulatory health centers). Health facilities included in the present study were belonging to categories 1, 4 and 5.

From November 2015 to April 2016, a cross-sectional study was conducted in all six public hospitals and in 19 out of the 26 private pharmacies in the 3 Health Districts (HD) of the Douala 5^e^ subdivision which is located in the Wouri Division, Littoral region. Douala V subdivision of Douala is an area of ~210 km^2^, making it the largest of the six subdivisions of the city of Douala, the economic capital city of Cameroon. This county shelters ~1,000,000 inhabitants who are divided into three HD according to the health division: Deido Health District (DHD), the “Cité des Palmiers” Health District (CPDH) and Bangue Health District (BHD). The six public hospitals were i) the Douala General Hospital (DGH), a 1^st^ category hospital at the top of the health pyramid, which provides specialized health care services and is considered as a reference hospital in the Central African sub-region; ii) “Cité des Palmiers” District Hospital (CPDH), a 4^th^ category hospital; iii) the 4^th^ Subdivision Medical Centers (SMC) belonging to the 5^th^ category of HF and namely Bepanda Medical Centers (BSMC), Cité SIC Medical Centers (CSicSMC), Bonamoussadi Subdivision Medical Centers (BoSMC) and Kotto Medical Centers (KSMC) (Fig 1).

Requests for research authorization were sent to all 26 private pharmacies in the district that are accredited by the National Order of Pharmacists of Cameroon (ONPC) for their location and for dispensing medicines and medical consumables. However, 5 of them did not respond positively and in 2 pharmacies, recruitment of individuals could not be done during the survey due to time constraints.

The questionnaire was administered in the study area following after a 1-week pretest to 15 people for each study population to assess: i) participant understanding and acceptance of the study and ii) to standardize and homogenize data collection across study sites. Interview questions were worded so as not to influence participants in their answers. The questionnaire was administered independently on the same day by two persons (a 7^th^ year pharmacy student-interviewer-1 and a 4^th^ year pharmacy student-interviewer-2) who interviewed participants within 10 mn of each other, to estimate inter-interviewer reproducibility. No one had received any antimalarial information sheets during these two interviews to minimize the risk of response bias.

After the interview and completion of the questionnaire, all customers were given an information sheets on antimalarial drugs, emphasizing the importance of rational use of antimalarial drugs as an infection control tool that can promote better self-management. This sheet allowed to have information on: What are the antimalarial drugs? What is antimalarial drugs resistance? What is "inappropriate" use of antimalarial drugs and what can physicians and other health professionals and the public do to fight to gain the abuse of antimalarial drugs consumption and antimalarial drugs resistance.

The study was conducted in accordance with the ethical guidelines for human’s research in Cameroon. The Ethics Committee of the University of Douala (N° CEI-UD/284/11/2015/T) approved the study and administrative authorization (N° 2672/AR/MINSANTE/DRSPL/BCASS) was obtained from the Littoral Health Delegation. Administrative authorization was also obtained from each HF and each pharmacy. Prior to enrollment and the administration of the questionnaire, subjects were informed of the purpose and procedure of the study (background, goals, methodology, study constraints, data confidentiality, and right to withdraw from the study), and a written informed consent was obtained from all participants who agreed to participate in the study in accordance with the Helsinki Declaration. To protect confidentiality and anonymity, we used anonymously collected data (encryption of identifiers by replacing names with codes) that are not linked to information that can identify the individual participant. Participation was voluntary, anonymous and without compensation.

### ii) Participants

The target population of the survey was all drug prescribers of public hospitals, outpatients attending the targeted HF and clients visiting private pharmacies located in the three HD covering the Douala 5^e^ subdivision.

#### Prescribers

A preliminary survey was carried out in all selected hospital services to determine the number of prescribers. All public HF were identified and stratified according to the different levels of the national health pyramid. A number of prescribers to be interviewed was then selected, which varied according to the different professional categories within each HF. Only prescribers who were available at the time of the study and who had given their verbal consent (in this case, the informed consent statement read to the participant to obtain consent was documented by marking the sheet with a code recognized by the investigator who obtained the consent) or written approval were included. Of the forty-eight (48) prescribers identified in the all selected HF, 41 (85.4%) have accepted to participate in the study.

#### Outpatients

Any person attending HD with fever or history of fever, with signs/symptoms suggestive of malaria infection, for whom a presumptive/confirmed diagnosis of UM was made, and who was willing to participate in the study by signing the informed consent form was enrolled in the study. Patients with other clinical symptoms not consistent with malaria infection or severe malaria were excluded from the study.

In 2011, a previous study conducted in two regions of Cameroon (Yaoundé in the Central Region and Bamenda in the Northwest Region) reported that among patients who were tested during their visit, 78% of those with a positive test result and 82% of those with a negative test result were prescribed or received an antimalarial drug [23]. Accordingly, and without information in Douala, a preliminary survey of 75 fever patients was conducted two months prior to the study and indicated that approximately 91.3% of those with a positive or negative test result were prescribed an antimalarial drug. To achieve the desired minimum sample size of N = 122 patients (using Cochran’s formula: N = Z^2^p(1-p)/d^2^ where Z = 1.96, is the confidence level statistic test at the desired confidence level and d = 0.05, is the accepted sampling error willing to be committed [30]), an average of 21 patients per selected HF were consecutively recruited between one and two weeks, depending on attendance.

#### Customers

Any person aged 15 years or older who requested the purchase of an antimalarial drug in the selected private pharmacy following a medical prescription, self-medication, advice from the pharmacist or pharmacy assistant, and who have gave their assent or consent by signing an informed assent/consent form was included in the study. The customers were asked verbally when they came to the pharmacy whether they would like to buy prescribed antimalarial drug or antimalarial drug for self-medication. If the answer was "no" they were excluded. If "yes", they will be approached to participate in the study.

The minimum sample size of malaria patients attending the selected private pharmacies was determined using the Cochran’s formula: N = Z^2^p(1-p)/d^2^ [30], where N is the required minimum sample size, Z is the confidence level statistic test at the desired confidence level (Z = 1.96), p: proportion of clients asking for antimalarial drugs (85%, based on our field experience), and d is the accepted sampling error willing to be committed (d = 0.05). This resulted in the minimum required number of 196 patients who were recruited consecutively.

### iii) Study questionnaire

Participants were asked to answer questions from the study questionnaire in one of the two national languages (English and French) that they understood best. They were open-ended interview questions (OEIQ) and closed-ended interview questions (CEIQ), including a single answer, and multiple choices questions.

For prescribers, data collection sheets were used to collect data on their socio-demographic characteristics, information on the continuing education, and attitudes toward malaria symptoms and treatment.

For patients attending HF, data collection sheets were used to collect data on their socio-demographic characteristics, and we sought to assess the reason for consultation, clinical diagnosis, paraclinical examination requested, and the medications prescribed. Patients’ medical records were also reviewed to complete the information required for the study.

Customers were asked to provide information on socio-demographic details. In the second part of the data, we sought to evaluate knowledge and attitudes towards malaria and the antimalarial drugs use. The third part focused on self-medication with antimalarial drugs or prescription of antimalarial drugs.

The questionnaire was administered by interview on 5 key points: socio-demographic data, knowledge and behavior towards malaria, prescribing, dispensing and prescribed drugs. All data were collected in the field.

### iv) Quality indicators used to evaluate antimalarial drug prescribing and dispensing practices

The following set of indicators were used according to drug prescribing practices or dispensing practices:

■ The proportion of prescribers who dispense an antimalarial drug before biological test result is available;
■ The proportion of prescribers who systematically dispense antimalarial drug to patients with fever;
■ The proportion of prescribers prescribing AS + AQ or AL as first-line treatment;
■ The proportion of patients with fever or history of fever
■ The proportion of patients who malaria test were prescribed but not performed it;
■ The proportion of patients who received an antimalarial before a biological test result was available;
■ The proportion of patients from whom ACTs were prescribed but with incorrect posology, dosage, formulation and duration of treatment;
■ The proportion of patients having received AS + AQ or AL as first-line treatment;
■ The proportion of customers who purchased ACTs without a medical prescription;
■ The proportion of ACTs among all antimalarials prescribed;
■ The proportion of customers who received education on drugs use;
■ The Proportion of customers who were prescribed ACTs in the correct dosage, posology, treatment duration of treatment and galenic formulation.

### v) Data quality assurance

Members or field teams were trained on the different activities including prospecting for HF, obtaining prescribers/patients consent, interviewing of prescribers/patients, and data reporting. The research team consisted of physicians, pharmacists, biologists, parasite experts and PhD/medical students. Completed questionnaires were reviewed by the principal investigators to ensure the reliability of the data collected. Data were entered into Excel and codes were assigned to different categories of each variable of interest to reduce typing errors.

### vi) Statistical analysis

Data were entered into an Excel spreadsheet and analyzed using the statistical package for social sciences v16 for Windows (SPSS, IBM, IL, USA) and GraphPad v5.03 for Windows (GraphPad, Inc., San Diego, USA). Data were presented as mean standard deviation (SD), median and percentage with confidence interval at 95% (95% CI) in tables and charts. Pearson’s chi square (χ^2^) test was used to compare qualitative variables while Student’s t-test, one-way analysis of variance, Mann-Whitney and Kruskal-Wallis tests were used to compare mean values between groups of interest where appropriate. Multivariate and univariate logistic regression analysis were used to identify the determinants of purchasing ACTs without a prescription (dependent variable). Odd ratios (ORs) were calculated to assess the strength of association between the dependent and independent variables. Variables with a *p-value* < 0.20 in the univariate logistic model were further used to build a multivariate logistic model. Significance was set at a *p-value* of less than 5%.

## Results

### Distribution of prescribers, patients and clients among the study participants

**Fig 2:**
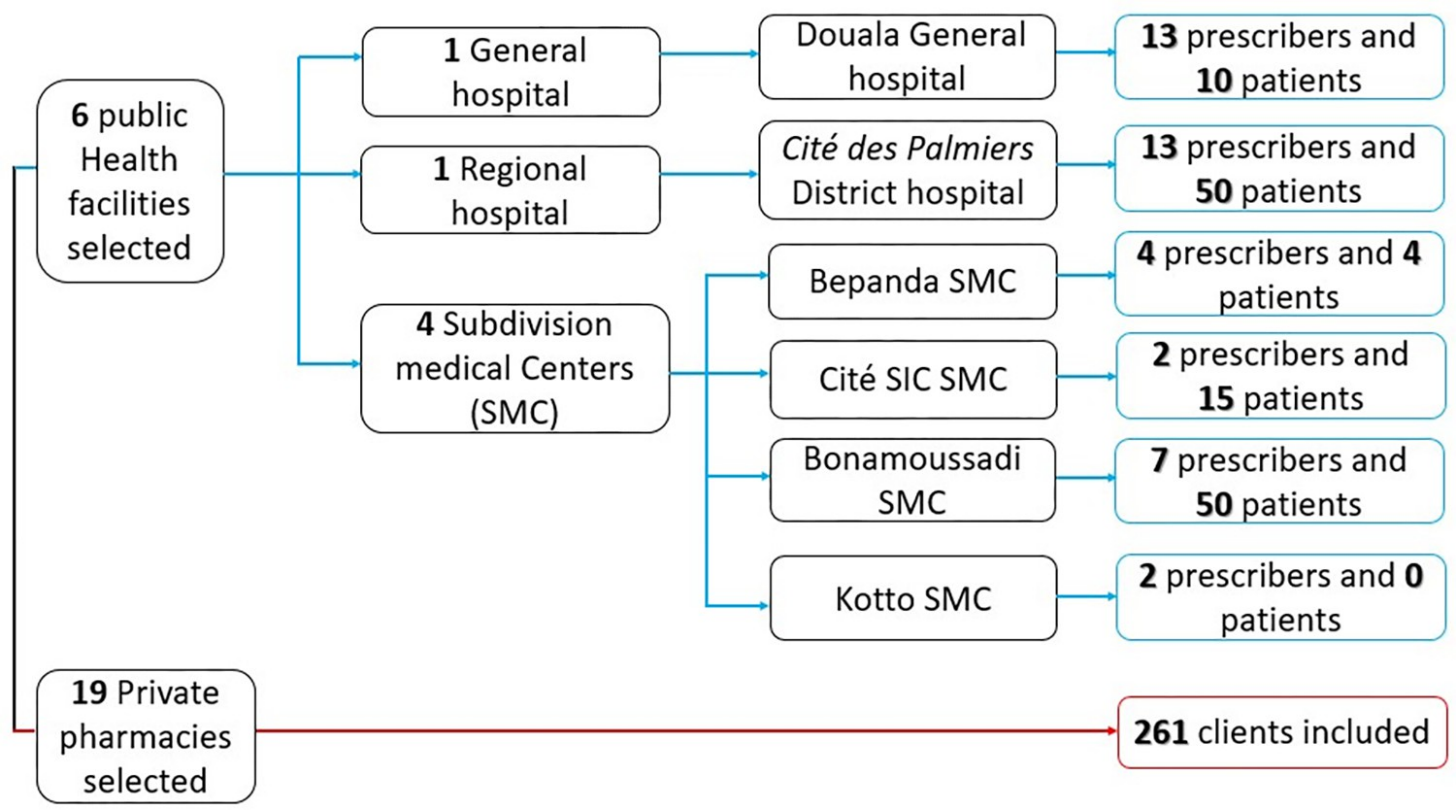
Flow diagram depicting the number of individuals included in the study.

Three populations were included in the study, namely drug prescribers and patients in HF and customers visiting private pharmacies. A total of 41 prescribers, 129 patients and 261 customers were enrolled in the study as shown in Fig 2.

### Drug prescribers

#### Demographical and professional profile of prescribers

Demographic and professional information of the health prescribers is shown in Table 1. The mean age of this population was 29.9±4.6 years old (min-max: 24–47 years). Prescribers from the KSMC were on average 31.0 years old, but no statistically significant difference was found between the mean age of prescribers from the different HF (Kruskal-Wallis test, *p* = 0.895). Most prescribers were female (63.4%), single (63.4%) and general practitioners (85.4%). More than one third (39.0%) had started their professional career 2–5 years ago. Approximately 73.2% had less than 6 years of experience, with experience ranging from 4 months to 35 years of experience.

**Table 1:** Details of health prescribers included in the study.

	Health facilities						Total (%) N = 41
Variables	DGH n = 13	CPDH n = 13	BepSMC n = 4	CSSMC n = 2	BoSMC n = 7	KSMC n = 2	
**Gender**							
Female	8	4	4	2	6	2	26 (63.4)
Male	5	9	0	0	1	0	15 (36.6)
**Mean Age (Sd), years**	30.5 (6.9)	29.6 (4.5)	29.8 (2.5)	29.0 (1.5)	29.8 (2.2)	31 (0.0)	29.9 (4.6)
**Marital status**							
Single	8	10	4	1	2	1	26 (63.4)
Married	3	3	0	1	5	1	13 (31.8)
Divorced	1	0	0	0	0	0	1 (2.4)
Missing data	1	0	0	0	0	0	1 (2.4)
**Training**							
Generalist	8	13	4	2	6	2	35 (85.4)
Specialist	5	0	0	0	1	0	6 (14.6)
**Years worked at facility**							
≤ 1	4	8	2	0	0	0	14 (34.2)
2–5	4	3	2	2	4	1	16 (39.0)
6–10	3	2	0	0	2	1	8 (19.5)
≥ 10	2	0	0	0	1	0	3 (7.3)

Data are number, unless otherwise indicated. DGH: Douala General Hospital; CPDH: “Cité des Palmiers” District Hospital; BepSMC: Bepanda subdivision medical center; CSSMC: “Cité SIC” subdivision medical center; BoSMC: Bonamoussadi subdivision medical center; KSMC: Kotto subdivision medical center

#### Knowledge of national treatment guidelines and training history

All prescribers were aware of the 2013 national guidelines on UM and SM management algorithm (Table 2). Half of them (57.5%) had received training on UM and SM management, mainly provided by the Ministry of Public Health (29.4%) and their employing HF (23.5%) (Table 2). On average, prescribers received their most training more than one year ago (12.9 ± 10.7 months). Prescribers from the CSSMC and KSMC have never received any training on national guidelines.

**Table 2:** Knowledge and training of 2013 national guidelines on malaria management.

	Health facilities						Total (%)
Variables	DGH n = 13	CPDH n = 13	BepSMC n = 4	CSSMC n = 2	BoSMC n = 7	KSMC n = 2	N = 41
**Knowledge of national guidelines**							
UM	13	13	4	2	7	2	41 (100)
SM	13	13	4	2	7	2	41 (100)
**Trained on management**							
UM	7	9	1	0	6	0	23 (57.5)
SM	7	9	1	0	6	0	23 (57.5)
**Mean period of the last training (Sd), months**	9.3 (4.2)	14.1 (13.5)	24 (0)	-	13.9 (12.7)	-	12.9 (10.7)
**Source of training**							
Ministry of Public Health	4	2	0	0	4	0	10 (29.4)
Employing health facility	2	2	1	0	4	0	9 (26.4)
Pharmaceutical laboratory	3	3	0	0	2	0	8 (23.5)
Personal	1	2	0	0	1	0	4 (11.8)
Scientific society/Organization	1	0	0	0	1	0	2 (6.0)
Media	0	1	0	0	0	0	1 (2.9)

Data are number, unless otherwise indicated. DGH: Douala General Hospital; CPDH: “Cité des Palmiers” District Hospital; BepSMC: Bepanda subdivision medical center; CSSMC: “Cité SIC” subdivision medical center; BoSMC: Bonamoussadi subdivision medical center; KSMC: Kotto subdivision medical center; SD: Standard deviation; SM: Severe malaria; UM: Uncomplicated malaria.

Although the prescribers from the BoSMC attended more training than the others, there was no significant difference (p>0.05) when comparing the different groups. For HF where prescribers attended training, the last training was on average 13 months ago (minimum: 14 days; maximum: 48 months).

Prescribers with more than 10 years of professional experience have attended an average of 5 continuing education courses. There is no significant difference between the average number of trainings attended and the number of years of experience.

#### Knowledge of malaria signs/symptoms

**Fig 3:**
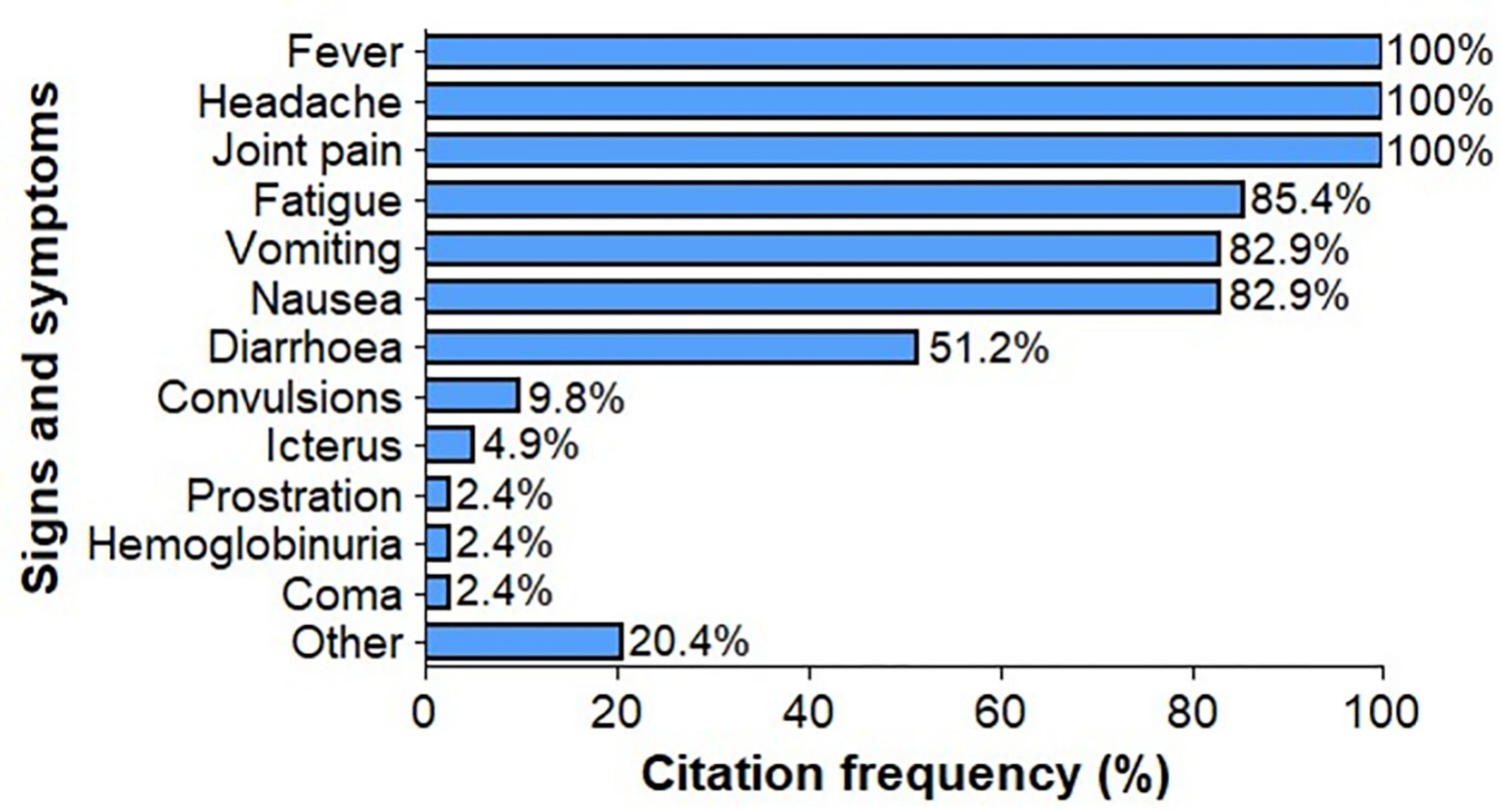
Citation frequency of signs/symptoms associated with malaria infection by health prescribers in their clinical practice.

The level of knowledge among prescribers was high, as evidenced by the large number of signs and symptoms reported to be associated with malaria infection (Fig 3). The most commonly reported signs/symptoms were fever, headache, joint pain, fatigue, vomiting and diarrhea. Fever, headache, joint pain to malaria symptoms alone account for 44.4% of the symptoms associated with malaria by prescribers.

#### Knowledge of prescribers on ACTs

All prescribers had heard of ACTs. Of the 41 prescribers interviewed, 14 (34.2%), 10 (24.4%) and 17 (41.4%) correctly named two, three, and more than three ACTs, respectively. Only one prescriber was able to name 5 ATCs. A total of six different therapeutic strategies, of which five ACTs were reported by the prescribers *viz*. AL (100%), DHA + PPQ (92.7%), AS+AQ (58.5%), ASMQ (36.6%), APPQ (7.3%) and ASSP (2.4%). Trimethoprim, an antibiotic, was associated with DHAPPQ in 9.8% of the prescribers.

#### Diagnosis and prescription practices regarding treatment of UM in children and adults

Not all prescribers waited for test results (i.e., RDT and PBF) before deciding whether to prescribe an antimalarial drug was requested. Indeed, only 39% of them waited for the PBF result and 68.3% for the RDT result before deciding whether or not to prescribe antimalarial drugs (Table 3), and no significant difference was observed between general practitioners and specialists.

**Table 3:** Diagnosis and UM treatment practices.

Variables, N = 41	n	%
**Waiting for test results before prescription of drug?**		
RDT, *yes*	28	68.3
PBF, *yes*	16	39.0
**Reasons for not waiting for test result**		
Signs/symptoms are sufficiently evocating for malaria	16	39.0
PBF is time-consuming	10	24.4
Lack of RDT/PBF reagents for diagnosis	9	21.9
Overwork in laboratory technicians	4	9.8
Concern on RDT sensitivity	3	7.3
High suspicion of self-medication by patients	1	2.4
**Do you systematically prescribe antimalarial drugs in case of fever and no parasitological test results?, *yes***	10	24.4
**If, yes give the reasons** *		
Malaria is endemic in Cameroon	6	60.0
There is a possibility to make clinical diagnosis	4	40.0
Malaria is the first consultation cause in endemic malaria area	1	10.0
Presence of fever	1	10.0
Fever is a vital emergency	1	10.0

Data are number and/or proportion (%), unless otherwise indicated. RDT: Rapid diagnostic test; PBF: Peripheral blood film.

*, Total sample size for this question was 10.

In general, the main reasons for prescribing antimalarial drugs regardless of test results were: signs/symptoms are sufficiently suggestive of malaria infection (39.0%), PBF is time-consuming (24.4%) and stock-outs of RDTs or PBF reagents (21.9%) (Table 3). Nearly 25% of them gave antimalarial drugs to patients with fever who had been tested for malaria. Of these, 60.0% said they did so because Cameroon is in a malaria endemic area.

**Fig 4:**
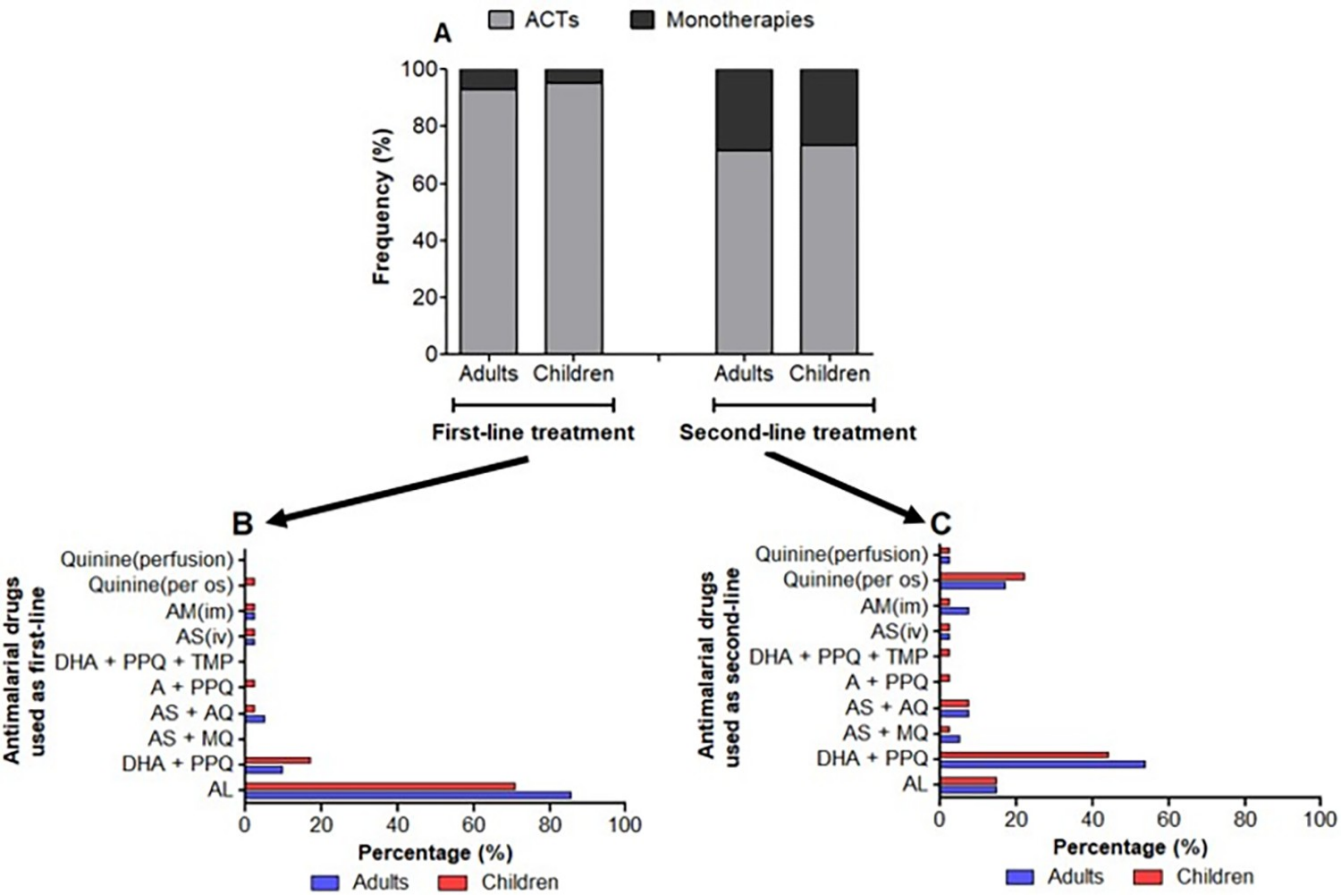
Drugs (A) used for treating UM as first-line (B) and second-line (C) treatment in adults and children. **Note.** AL: Artemether + Lumefantrine; DHA+PPQ: Dihydroartemisinin + Piperaquine; AS + MQ: Artesunate + Mefloquine; AS +AQ: Artesunate + Amodiaquine; A + PPQ: Arterolane + Piperaquine; DHA + PPQ + TMP: Dihydroartemisinin + Piperaquine + Trimethoprim; IV: Intra-venous; IM: Intramuscular.

ACTs were prescribed as first- and second-line treatment in ≥ 90% and ≥ 72% of UM cases, respectively, regardless of patient’s age (Fig 4). Monotherapies with quinine, AM and AS were also prescribed for febrile patients. AL and DHA + PPQ were mainly prescribed as first-line treatment in both adults and children, while DHA + PPQ and quinine (*per os*) were mainly prescribed as second-line treatment. AS + AQ accounted for 4.9% of first-line prescriptions in children and 2.6% in adults.

In addition, the posology and duration of treatment with these drugs according to the patient’s age were respected, as recommended by national guidelines. Regarding the quality of the prescription of antimalarial drugs used as first-line treatment for UM, the dosage of ACTs was correct in 73.0% (27/37) of the prescriptions in children and 92.1% (35/38) in adults. For AL, the main drug prescribed, the correct dosage was found in 77.1% (24/31) of children and 89.7% (26/29) of adults. The duration of treatment of ACTs reported by prescribers was correct for both adults and children, with 95.1% (39/41) of prescribers adhering to the prescribed duration of treatment (Fig 4).

Regarding the quality of prescribing of second-line antimalarials for UM, the dosage of ACTs was correct in 60.0% (18/30) and 86.7% (26/30) of prescriptions for children and adults, respectively. DHA + PQP doses were incorrect in 40.0% of cases in children and 5.6% of cases in adults. AS + AQ doses were incorrect in 1/3 of patients and oral quinine doses were incorrect in 83.3% of pediatric prescriptions and 25.0% of adult prescriptions. The duration of treatment with ACTs was appropriate for both adults and children, and 93.5% of prescribers adhered to the prescribed duration of treatment (Fig 4).

A total of 12 prescribers argued that they also prescribe non-ACT drugs for the treatment of UM. The reasons were as follows: Similar efficacy to ACTs (n = 2), in case of ACT failure (n = 1), allergy/intolerance to ACTs (n = 1), ACT stock-outs (n = 3), easier to prescribe non-ACTs (n = 1), suspected ACT resistance (n = 1), patient request (patient was unwilling to take drug) (n = 1), non-ACT drugs more readily available (n = 1), low cost of non-ACTs/low-income of patients (n = 3) and other (n = 3).

Paracetamol was the main non-antimalarial drugs prescribed concomitantly with antimalarials. Antibiotics such as TMP were also prescribed together with antimalarials, especially DHA + PPQ combinations. All the prescribers reported waiting for complete blood counts results before prescribing anti-anemia drugs.

### Patients in health facilities

#### Demographical, clinical and paraclinical profile

A total of 129 of outpatients were included in the study, 62.0% of whom were women, resulting in a female-to-male ratio of 1.63. The mean age of the patients was 18 years (range: 1 month to 84 years). Patients from Bepanda SMC were on average older (38.8 ± 15.9 years old) than those from other HF (Kruskal-Wallis test, *p* = 0.002). The majority (100, 78.1%) of these patients resided in Douala 5^th^ sub-division, while 21 (16.4%) and 6 (4.7%) resided in Douala 3^th^ or 1^st^ sub-division, respectively.

Eighty-nine (69.0%) were seen by a general practitioner while the rest of the patients were seen by a specialist (n = 36, 27.9%), a 7^th^-year medical student (n = 3, 2.3%) or nurse (n = 1, 0.8%).

#### Information on clinical examination, malaria diagnosis and treatment

**Fig 5:**
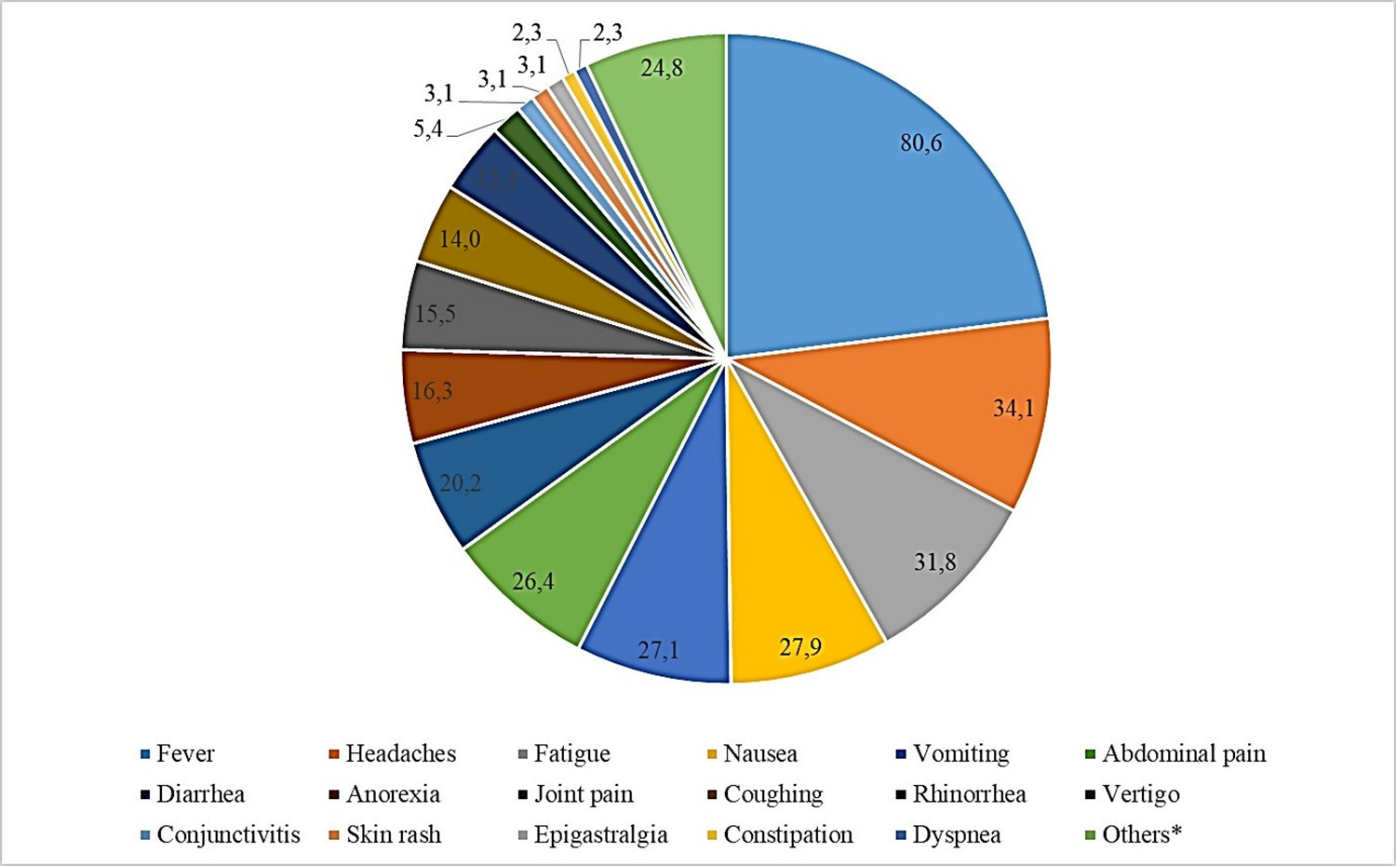
Prevalence of symptoms presented by patients and reported in the patient record. **Note.** *, oral bitterness, angina, dysuria, edema, nocturnal sweating, nocturnal chills.

Fever (80.6%), headache (34.1%), and fatigue (31.8) were the top three reasons for patient attending HF among reported symptoms (Fig 5).

Of the 129 outpatients enrolled in the study, 119 (92.2%) were classified as having malaria. The term "UM" was reported in 52.8% (68/129) of the medical records. Different terms were also used to describe the clinical diagnosis of malaria, including "malaria" in French (17.1%) or in English (1.6%), "simple malaria" (51.7%), "maltreated malaria" (3.1%), "maltreated simple malaria" (0.7%), "simple malaria attack" (3.9%) and "malaria attack" (6.2%). In 14.7% of patients, no clinical diagnosis was given in the medical records.

Prescribers recommended a biological test in 99.2% (128/129) of patients, and the most commonly requested test was the thick blood smear (122/128, 95.3%), while RDT was requested in 51.6% (66/128) of patients. However, malaria tests were performed in only 60.1% (78/128) of patients for whom a test was prescribed. Approximately 34.9% of patients had a positive RDT and/or PBF and 6.2% had a negative RDT and a positive PBF.

ACTs were predominantly (94.5%) prescribed to patients, with AL (71.0%) and DHA + PPQ (21.7%) being the most commonly prescribed ACTs. No prescriptions included AS + AQ (Table 4).

**Table 4:** Diagnosis and treatment findings.

Variables, N = 129	n	%
**Malaria test asked by the prescriber**		
No	**1**	0.8
RDTonly	6	4.6
PBF only	62	48.1
RDT+PBF	60	46.5
**Malaria test asked and performed**		
RDT performed?, *yes*	36	54.5^$^
PBF performed?, *yes*	74	60.7^&^
**RDT/PBF Results**		
RDT (+) and/or PBF (+)	45	34.9
RDT (-) and/or PBF (-)	65	50.4
RDT (-) and PBF (+)	8	6.2
Missing data	11	8.5
**Patients having received an antimalarial treatment**	73	56.6
**Patients having received a treatment before getting test results** ^**§**^	42	57.5
**ACTs prescribed** ^**§**^	69	94.5
**Drugs prescribed** ^**#**^		
AL	49	71.0
DHA+PPQ*	15	21.7
A+PPQ	4	5.8
AS+MQ	1	1.4
AMim	4	5.8

Data are number and/or proportion (%). AL: Artemether+Lumefantrine; DHA + PPQ: Dihydroartemisinin+Piperaquine; AS + MQ: Artesunate+Mefloquine; A + PPQ: Arterolane+Piperaquine; AMim: intramuscular Artemether; RDT: Rapid diagnostic test; PBF: Peripheral blood film.

^$^, Percentage was computed using 66 as denominator (number of patients with RDT test asked).

^$^, Percentage was computed using 122 as denominator (number of patients with PBF test asked).

^§^, Percentage was computed using 73 as denominator (number of patients having received an antimalarial drug)

^#^, Information on ACTs prescribed was missing for 4 patients

*, Trimethoprim (antibiotics) was co-administered with DHA and PPQ in 2 patients.

Regarding the appropriateness of prescribing according to antimalarial drugs, AL was the only antimalarial drug for which the dosage, quantity, dosage and duration of treatment were not correct in 2, 1.14 and 5 patients respectively.

Interestingly, of the 73 patients (56.6%) who had received antimalarial treatment, more than 57% had received it before test results (Table 4).

#### Rationalization of ACTs usage among patients

**Fig 6:**
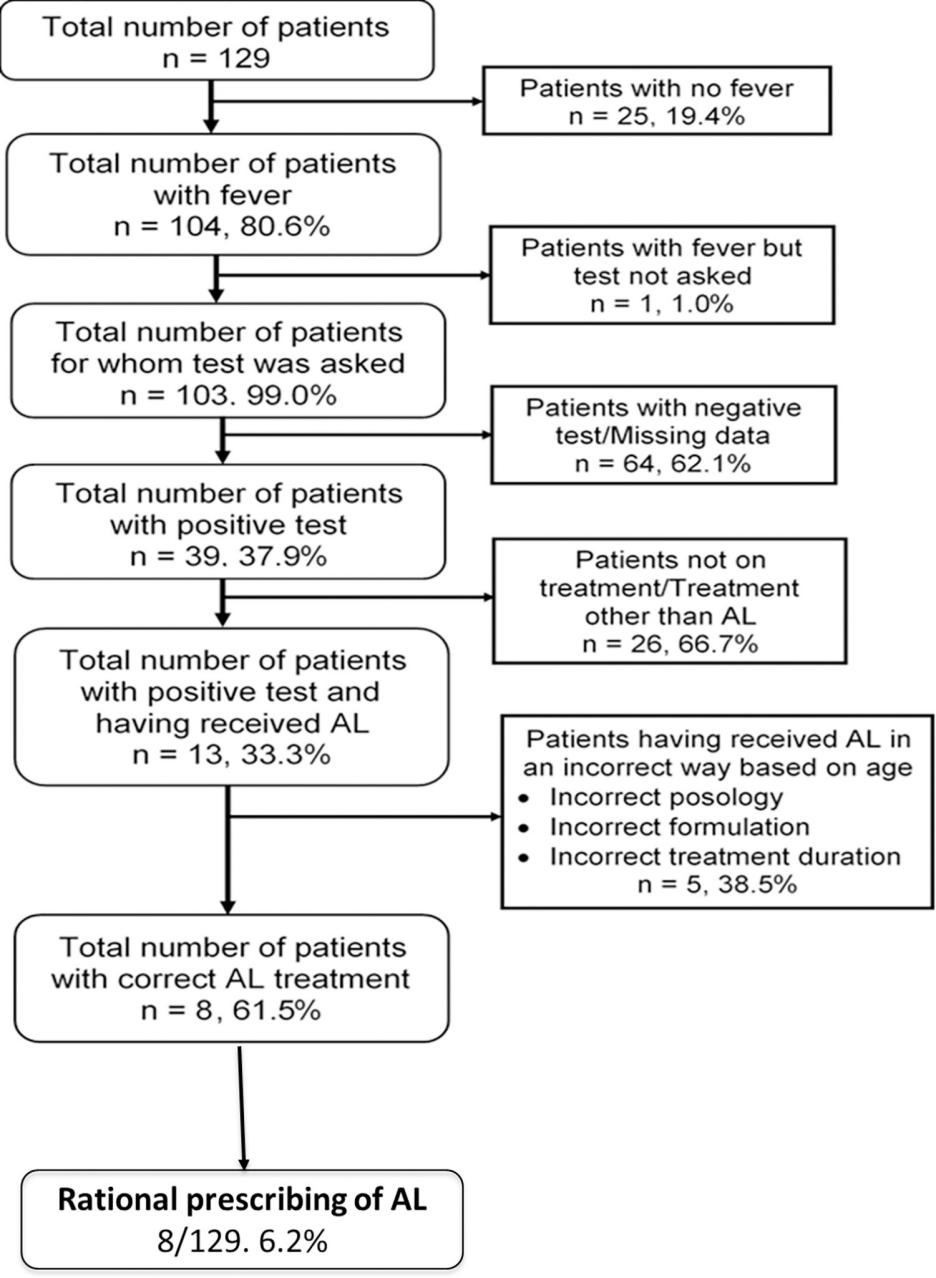
Findings on rationalization of AL usage in patients in health facilities in the Douala V subdivision of Douala.

Based on the six criteria we used to assess the rationalization of AL use. AL were administered correctly in 8 out of 13 patients (61.5%) who received this ACT in a rational manner (Fig 6). However, of the 129 patients who presented to the heath structure with suspected malaria, only 6.2% (8) were treated rationally according to the prescription.

In addition, as noted in the section on prescribers, few patients had received an antimalarial drug prior to malaria testing: 29.7% and 7.7% of them had received treatment prior to RDT and PBF, respectively.

### Customers attending private pharmacies for antimalarial drugs

#### Demographical profile

Of the 760 customers who visited the pharmacies during the study period, 266 (35%) purchased antimalarial drugs, of whom, 261 (98.1%) agreed to participate in the study.

The mean age was 33.7 years (interquartile range: 27.1–39.1), with a male to female ratio of 1.21/1. There was a statistically significant association between gender and educational level. Men had mostly completed university studies (55.2%) compared to women who had mostly completed high school (42.4%) (Chi square test, *p* = 0.018).

#### Information source on malaria, Knowledge and perception about antimalarial drugs

This study reported several sources of information from which respondents learned about malaria and prevention methods. Some respondents reported more than two sources of information. The main sources of information were radio and television (58.5%), mass communication (23.0%), and educational talks and theater (20.5%). A few respondents were aware of malaria and prevention methods through interpersonal communication (1.2%).

Overall, 204 (78.2%) participants claimed to know what an antimalarial drug is, compared to 21.8% who did not know. Of those who claimed to know what an antimalarial drug was, only 3.9% were able to define an antimalarial drug as an anti-parasite drug. The rest of the answers were misconceptions or no answer: 48.0% said the antimalarial was used against bacteria, 19.1% defined it as a "cure all", 3.9% as an antiviral, 2.5% as an antipyretic, and missing data accounted for 22.6%.

The majority of people (246 respondents, 94.3%) said that antimalarial drugs could elicit side effects while the rest of participants said that these drugs do not cause side effects (1.1%) or had not any idea about the question (4.5%). The most common side effects reported by responders were allergy (34.0%), dizziness (30.7%), and digestive disorders (26.7%). Some rare side effects (8.6%) were also reported: tinnitus, hot flushes, headache, skin rash, gastralgia, hyperthermia, insomnia and halitosis.

Two hundred and sixteen respondents (82.8%) stated that the use of an antimalarial drugs is dangerous, this is the main reason why only health workers are authorized to prescribe them. Other reasons, including the high proportion of counterfeit drugs on the market, the development of drug resistance in parasites, and the misadministration of drugs before or without biological testing, were also cited by respondents to justify the dangers of these drugs.

#### Purchasing antimalarial drug trends, associated factors and malaria symptomatology

Most of the antimalarial drugs were purchased for the treatment of adults (144/261, 55.2%), the rest for children and adolescents. About 53.5% of the responds reported that they bought antimalarials for their own treatment, while 46.5% bought them for a relative. The dispensing of antimalarial drugs was mainly done by pharmacy assistants (53.8%).

More critically, 69.4% (181) of the clients bought antimalarial drugs without a medical prescription and this self-medication was driven by the advice of the pharmacy assistant (76.8%) and senior pharmacist (23.8%). Self-medication with antimalarial drugs was more common in males than in females (OR = 1.77, 95% CI: 1.04–4.00, *p* = 0.036) and was twice more frequent in adults than in children (OR = 2.09, 95% CI: 1.22–5.80, *p* = 0.007).

Fever (85.1%), fatigue (49.0%), headache (43.3%) and joint pain (37.6%) were the most common signs/symptoms reported in patients’ medical records. The mean time from symptom onset to medical consultation (prescription) was 3.4 days (range: 1–8 days). The mean time from symptom onset to over-the-counter purchase of drugs without prescription was 3.4 days. No statistically significant difference (*p* = 0.634) was found between children/adolescents and adults for these two mean durations.

In addition, patients with a prescription took five times longer to attend health care facility after the onset of symptoms than those who self-medicated, and this difference was statistically significant (OR: 5.34, 95% CI 2.94–9.74, *p* < 0.0001).

#### Malaria diagnosis

Malaria diagnosed in 49 (18.8%) of the patients before the purchase of the antimalarial drug. The result of malaria test was available in 43 patients. Biological test for malaria was performed in 23.9% of children/adolescents versus 14.6% of adults (*p* = 0.056). The type of diagnostic test performed was known in 43 out of these 49 patients, and consisted of PBF (38 patients, 88.4%) and RDT (10 patients, 23.3%). Both RDT and PBF were performed in a few patients. PBF and RDT were both performed in some patients. The result of the malaria test was unknown (not recorded in the medical record) in 23.3% (10/43) of the patients, while the tests were all positive in the remaining patients (87.9%, 29/33).

#### Dispensation and rationalization of ACTs

ACTs accounted for 90.0% of the antimalarials purchased in pharmacies, of which 30.1% were recommended by prescription. The rest of drugs included artemether, quinine and SP.

**Fig 7:**
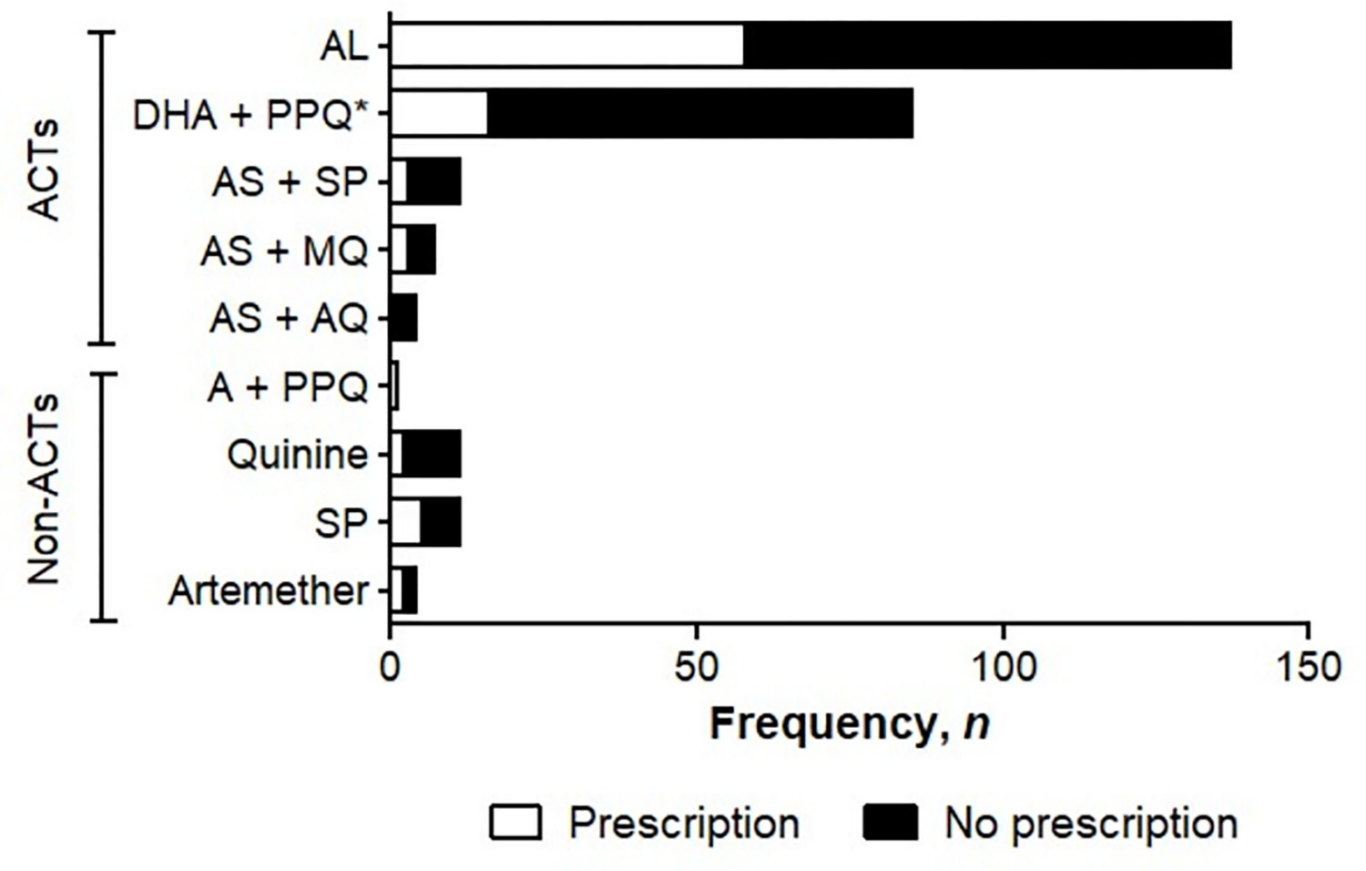
Antimalarial drugs purchased in pharmacies. **Note.** ACTs: Artemisinin based combination therapies; AL: Artemether + Lumefantrine; DHA + PPQ: Dihydroartemisinin + Piperaquine; AS + MQ: Artesunate + Mefloquine; A + PPQ: Arterolane + Piperaquine; *: Trimethoprim (antibiotics) was co-administered with DHA and PPQ in some patients.

Irrespective of the type of drug, the most commonly purchased combinations were AL (50.6%) and DHA + PPQ (29.5%). The same pattern was observed among those with or without a prescription (Fig 7). As observed in the previous section on HF, AS + AQ, which is recommended as first-line treatment according to national guidelines, accounted for only 1.6% of all ACTs purchased and was purchased only by customers without a medical prescription.

The galenic formulation of ACTs was correct in 100%, the dosage in 97.0%, and the posology in 75.7%.

**Fig 8:**
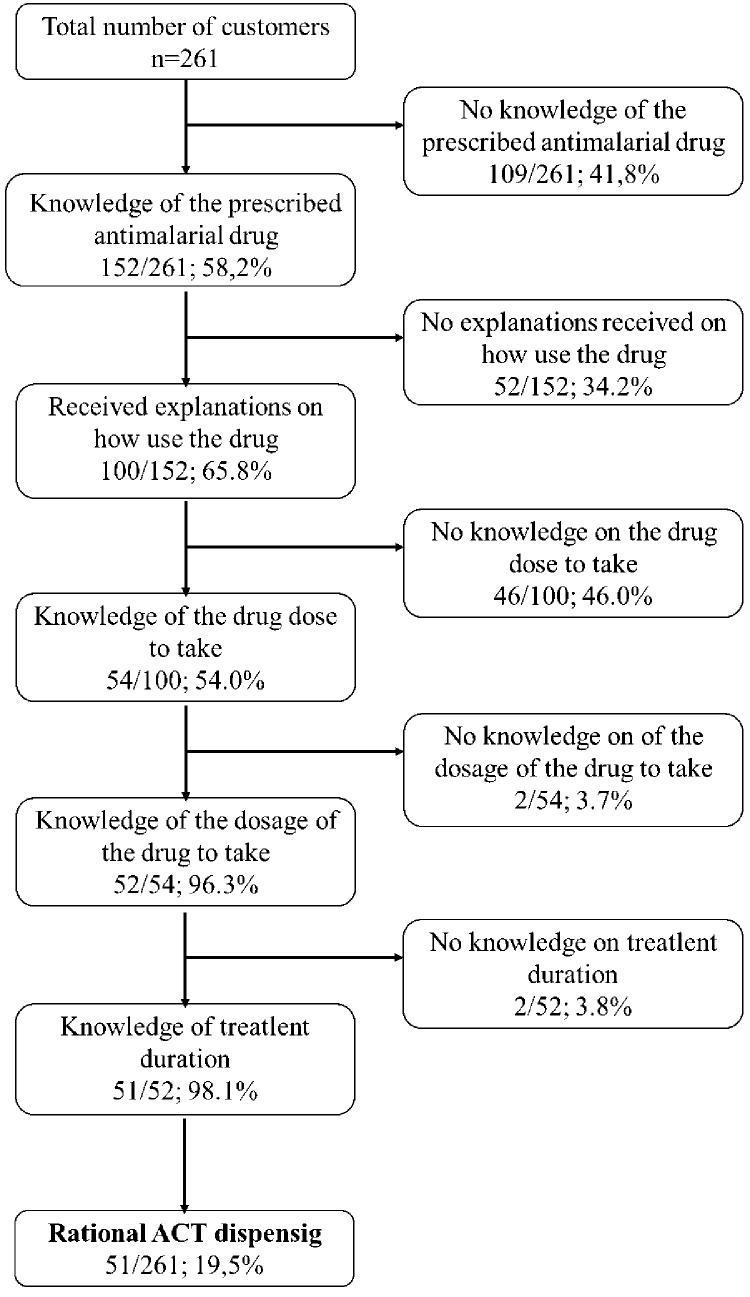
Global evaluation of antimalarial drug dispensing in private pharmacies in the Douala V subdivision of Douala.

A total of 152 (58.3%) of 261 customers claimed to know the antimalarial drug they had purchased. Among them, 100 received explanations on how to use the drug should (i.e., posology and duration of treatment) (Fig 8). On detailed analysis of these 100 clients, 54 of them gave correct answer on the drug dose to be taken, 52 on the posology and 51 on the duration of the treatment of the purchased antimalarial drug. Considering all these evaluation criteria, the rationalization of dispensing was observed in 51 (19.5%) customers who came to buy the antimalarial drug (Fig 8).

## Discussion

ART resistance constitutes a serious concern for malaria control in Southeast Asia, which is the only region where ART resistance is confirmed and widespread. The main concern is the potential emergence and/or spread of ART resistance to sSA, which accounts for the majority of the malaria burden. Recent studies reporting the local emergence of ART-resistant parasites in Rwanda and Uganda have raised this concern [9, 10, 14]. Therefore, rational use of ACTs is of paramount importance to prevent ART resistance in malaria-endemic areas. The aim of this study was to evaluate the prescribing and dispensing practices of ACTs in the Douala 5^e^, in Cameroon, an urban area with a high malaria burden.

This study reported that only 58.5% of prescribers had received continuing training in the management of simple and severe malaria. This prevalence is higher than that reported by Mangham et al. in 2009, who found that in public health facilities in Cameroon (Central and North-West regions) and Nigeria (Enugu State), 47.9% and 31.6% of prescribers, respectively received continuing training on the management for malaria [29]. Ongoing training organized by the Ministry of Public Health (29.4%), health facilities (26.4%), and pharmaceutical laboratories (23.5%) were the main channels through which prescribers received refresher training on malaria and its treatment. Another study conducted in 2005 in Yaoundé, Cameroon, reported that medical representatives were the main source of information on antimalarial drugs for prescribers (70.5%), followed by health workers (12.9%) and the Ministry of Public Health (6.8%) [31]. The results of the current study suggest that over the years, the Ministry of Public Health has made significant efforts to raise awareness and train medical staff about malaria and its management.

ACTs were mainly suggested (prescribers), prescribed (patients) and purchased (customers), outlining the impact of the information strategies implemented by the Cameroonian government since the decision to use ACTs in 2004. This finding is consistent with those from previous reports in Cameroon [23, 24, 26, 29] and other African countries [32, 33]. Some ACTs (DHA + PPQ, AS + SP) that were not adopted at the time of the study were prescribed or purchased. These drugs are recommended by the WHO and have been shown to be as effective as the adopted ACTs (i.e., AS + AQ, AL) for the treatment of UM in Cameroon [34, 35]. DHA + PPQ was recently included in the updated national guidelines. Some monotherapies have been proposed, prescribed and purchased for the treatment of UM, namely quinine, artemether and AS. In Cameroon, these drugs are recommended for the treatment of SM via parenteral routes such as intramuscular or intravenous. These non-ACTs were preferred by some prescribers despite their knowledge of national treatment guidelines. This study showed significant use of ACTs (94.5% vs 5.5% for monotherapies) compared to rates reported in other studies conducted between 2006 and 2014, which showed that ACT prescription rates increased from 46% to 78% [26, 36]. Taken together, these findings show an increase in the use of ACTs in the treatment of UM and, consequently, a good compliance of prescribers with recommendations of the Ministry of Public Health, although there are still cases of rampant use of monotherapies. Reasons such as stock-outs of ACTs were given to explain these prescribing practices, highlighting the importance of environmental context in making prescribers’ decisions [29, 37].

Antimalarial treatment was given (patients) or prescribed (prescriptions) while the biological test result was still unknown. The percentage of patients receiving antimalarial treatment without biological confirmation was 57.5%, which is similar to that reported in Benin (53%) [38], but higher than that reported in the Democratic Republic of the Congo (9%) [39] and lower than that reported in Gezira State, Sudan (96.5%) [32]. Prescribers justified starting treatment before the results of biological tests for several reasons: i) most patients did not immediately undergo the required biological tests and therefore, if they were sent home without treatment, symptoms could worsen and case management could be even more costly. Therefore, the prescribers started antimalarial treatment when the patients had fever (clinical signs of malaria). The second reason given was that most patients had used drugs in self-medication before coming to the hospital, which could result in negative RDTs and/or tick blood smears. The lack of RDTs or tick blood smears reagents stock-outs in some of the study sites, the waiting time for tick blood smear results considered too long by prescribers, and the fact that Cameroon is located in a malaria endemic area are other arguments used to explain the high proportion of presumptive treatment of UM in our study. The WHO recommends that whenever possible, "*in all settings*, *clinical suspicion of malaria should be confirmed with a parasitological diagnosis* " [40]. Thus, additional efforts should be made to strengthen the capacity of health facilities to provide parasitological diagnosis to patients as the adherence to performing malaria testing is a key component for the rational use of ACTs.

Despite the fact that we were unable to obtain information on the test result, this finding may imply that ACTs may have been supplied to a number of patients with negative results, as previously shown in Cameroon [26]. The adherence of prescribers to the sensitivity of signs/symptoms presented by patients was one of the reasons given by them. This finding is in line with Worges *et al*. who found that headache was a significant predictor of antimalarial drug treatment or prescription among untested Zambian patients [41]. This clinical sign was among the top three of signs/symptoms reported by patients in our study. Another explanation may be that some prescribers still use the concept that "*a negative result does not rule out malaria*". We have found that few prescribers were reluctant to adhere to test results because of concerns about sensitivity and self-medication.

Even when prescribed and administered to patients with a positive test result, ACTs were not administered correctly, as few cases of incorrect posology, dosage, formulation and duration of treatment were reported for ACTs, especially AL, in the study. The errors were mainly with the powder forms of AL for oral suspension in children as previously reported by Elmannan et al. [32], who found that only 72.5% of AL dosages were correct. Prescriptions for powdered AL for oral suspension in children, regardless of the level of training of the prescriber, indicated once-daily administration for three days instead of dividing the doses into two doses for three days. These errors can lead to treatment failure or the development of resistance, or put patients at risk of overdose. This suggests the need for government training campaigns, as such malpractices expose patients to ACT side effects, malaria complications and death. At the population level, this increases the chances of the parasite developing resistance to ACTs.

AL (76.2%) and DHA + PPQ (46,0%) were mainly recommended (prescribers) and prescribed (patients) as first-line treatment and second-line treatment of UM respectively, while AS + AQ was rarely prescribed (2.6% in adults and 4.9% in children). AS + AQ was recommended as first-line treatment for UM in Cameroon at the time of the current survey, so this finding was not in line with the 2013 national guidelines. Such results have also been reported in previous studies in the Centre, Littoral and Southern regions of Cameroon [31, 36, 42]. The predominant use of AL and DHA + PQP could also be justified by the low prescriber awareness of AS + AQ among prescribers compared to the other two molecules. We found that AL was the most known ACT by prescribers (32.5%), followed by DHA + PQP (30.2%), and AS + AQ (19.0%) as reported in a previous study in Yaoundé where AS+MFQ was the most known ACT by prescribers (9.9%) followed by AL (7.6%) and AS + AQ (6.8%) [42]. During the study period, only 3 ACT specialties were available from private wholesaler-distributors in Yaoundé: Arsucam®, Coartem® and Artequin® [42]. During the present study, 8 ACTs were available and sold in private pharmacies in Douala V subdivision. Low prescription rate of AS+AQ was also reported in Equatorial Guinea and Uganda where this ACTs is adopted as first-line treatment of UM during the same period [43, 44]. There is a belief among patients and health care providers that this combination, especially AQ, causes serious side effects (mainly fatigue) as previously reported by Sayang *et al*. [31, 42]. We did not address the raisons for the low prescriptions of AS + AQ, but this belief about AS + AQ is empirically supported by some studies in Cameroon. Apinjoh *et al*. reported side effects (mainly fatigue, dizziness and cough mainly) in Cameroonian children treated with AS + AQ for UM [35]. Another study reported higher rate of adverse events in patients treated with AS + AQ compared to those treated with AL [33]. Other studies reported a high prescribing rate of AS + AQ according to national guidelines of the respective government [39]. In addition to ACTs, 5.5% of prescriptions for UM included monotherapies, mainly AMim, based on the personal request of patients who were reluctant to swallow the tablets.

Antimalarial drugs, especially ACTs, were purchased without a medical prescription (malpractice) and dispensed mainly by pharmacy assistants. This malpractice has been previously reported in Cameroon and other countries [38, 45]. In general, pharmacy assistants did not received any specific training on when drugs should be dispensed. This finding outlines the need to address the training of pharmacy assistants to limit the dispensing of antimalarial drugs without a prescription. Even when drugs were dispensed without a prescription, few clients were not given any information on how to use the drugs. This may put them at risk of experiencing side effects, worsening health status and deats due to misuse of the drugs [45]. Similarly, as noted above at the population level, self-medication increases the antimalarial drug pressure and the risk of emergence and/or selection for ART-resistant *P*. *falciparum* isolates.

Rational use of medicines is one of the priorities of this therapeutic optimization. Defined by the WHO in 1985, it consists of "prescribing the most appropriate product, obtained on time and at an affordable price for all, delivered correctly and administered at the appropriate dose and duration". Worldwide, more than 50% of medicines are prescribed, distributed or sold inappropriately [46]. At the same time, about one-third of the world’s population does not have access to essential medicines and 50% of patients do not take them correctly [47]. The consequences are ineffectiveness, toxicity, increased delays in seeking quality care, development of resistance, and economic waste for both the family and the community/state. More than 93% of the prescriptions reviewed in this study showed a high rate of prescribing irrationality in the selected public health facilities. Numerous studies have been conducted worldwide in in East Africa [32, 48, 49], in West Africa [50–52], in Central Africa in Cameroon [23, 25, 26, 28] and, have shown the difference in the rate of prescribing rationality varying between 26% and 54.6% and the sample size between 71 and 2,519 for patient population and between 54 and 196 for prescriber population. In Asia, a very high rational prescribing rate of 94% was found in a study conducted in Afghanistan in Asia [53]. In these studies, the criteria for assessing the rational management of patients with UM were any patient seen in consultation for fever with a positive thick blood smear result and prescribed the recommended antimalarial drug in first line at the right dosage. In our study, in addition to these 4 criteria, we also considered the prescriber’s request for the patient to perform the RDTs and/or tick blood smears by the patient, the appropriateness of the prescription in terms of dosage, galenic form and duration of the treatment.

More than 80% of the dispensing reviewed showed a high rate of dispensing irrationality in the selected private pharmacies in the Doula 5^e^ subdivision. In addition, 26.2% of patients were not explained how to use their antimalarial drug. In a previous study in Sudan, 51.1% of patients were not well informed about their medication at the time of dispensing [32]. Therapeutic education of patients is to be encouraged as it improves their adherence to prescribed treatments, leading to better therapeutic outcomes as shown in some studies [44, 54].

This study should be interpreted in light of its limitations, as it was conducted in one of the six subdivisions of Douala, and thus the findings do not necessarily reflect the picture in the whole city.

## Conclusions

The present study first outlined a high level of knowledge about malaria and national guidelines for the management of UM among drug prescribers. However, there was a gap between this knowledge and their routine practice. Indeed, (i) the therapies recommended at the time of the study were not systematically used, (ii) ACTs were often prescribed without guidance from malaria test results. Second, these findings were confirmed by interviews with patients attending HF of drug prescribers. Thirdly, ACTs were mainly dispensed in private pharmacies without medical prescription. Information on ACTs dispensed was not systematically provided to customers, and information on dosage, posology, duration of treatment and galenic formulation were wrong in some medical booklets. Therefore, it is very critical to develop, implement and scale up control strategies tailored to health practitioners and the community regarding the use of ACTs in Cameroon. As we are also aware of the fact that the data may not be consistent with current practice and without recently published study in rationalizing the use of ACTs for treatment of UM, other studies will need to evaluate this rationalization.

## Abbreviations

ACT: Artemisinin-based combination therapy
A: Arterolane
AL: Artemether + Lumefantrine
AQ: Amodiaquine
ART: Artemisinin
AS: Artesunate
BHD: Bangue Health District
CI: Confidence interval
CPHD: “Cité des Palmiers” Health District
DHA: Dihydroartemisinin
DHD: Deido Health District
IM: Intramuscular
IV: Intra-venous
MD: Medical doctors
MQ: Mefloquine
NA: Not applicable
NW: North-West
OR: Odds ratio
PPQ: Piperaquine
PBF: Peripheral blood film
PY: Pyronaridine
RDT: Rapid diagnostic test
SD: Standard deviation
SMC: Subdivision medical center
SP: Sulfadoxine-Pyrimethamine
sSA: sub-Saharan Africa
TMP: Trimethoprim
UM: Uncomplicated malaria
SM: Severe malaria
SW: South-West
WHO: World Health Organization
